# Post treatment lower limb lymphedema in carcinoma cervix

**DOI:** 10.3332/ecancer.2025.1879

**Published:** 2025-03-20

**Authors:** Shreekant Dadheech, Kaustubh Burde, Pariseema Dave, Ruchi Arora, Chetna Parekh

**Affiliations:** Department of Gynecological Oncology, G.C.R.I., Ahmedabad 380016, Gujarat, India

**Keywords:** carcinoma cervix, lymphedema, quality of life

## Abstract

**Purpose:**

To determine the incidence of post-treatment lower limb lymphedema in cases of carcinoma cervix and its correlation with demographic and treatment-related factors.

**Method:**

A retrospective observational study conducted over a duration of 2 years.

**Inclusion criteria:**

Patients with carcinoma cervix who have completed their primary treatment, which included surgery followed by radiotherapy (RT) or concurrent chemo-radiotherapy (CCRT) or CCRT + Brachytherapy. All patients of carcinoma cervix were screened in the OPD during 3-monthly follow-ups post-completion of their treatment.

**Results:**

In our study, we analysed 100 patients, of whom 15 developed lymphedema. The odds ratio was greater than 1 for external beam RT (OR-1.03), age of the patient (OR- 1.04) and stage of the disease (OR– 1.4). Thus, an association was found between lymphedema and the age of the patient, stage of the disease and external beam RT. No association was found between lymphedema and surgery or body mass index (BMI) of the patient.

**Conclusion:**

Our study found an association between lower extremity lymphedema post-treatment in cervical cancer with age, International federation of Gynaecology and Obstetrics stage and method of treatment. However, BMI was not associated with lower limb lymphedema post-treatment.

## Background

Carcinoma cervix accounts for 17.7% of all new cancer cases in females in India, according to GLOBOCAN 2022 [1]. Cervical cancer is primarily treated with surgery, radiation or both. Part of the treatment includes the removal of pelvic lymph nodes or irradiation of the same, which disrupts and obstructs lymphatic drainage in the lower limb leading to lower limb lymphedema. Five-year survival rates are 91.6% for stage IB1, 83.3% for stage IB2, 76.1% for stage IB3, 40.7% for stage IIIA, 41.4% for stage IIIB, 60.8% for stage IIIC1 and 37.5% for stage IIIC2 [2]. Improving quality of life is a priority, given the better survival rates in carcinoma cervix post-treatment.

Lymphedema is a chronic, complex condition affecting approximately 20 million people worldwide, causing significant discomfort, morbidity and financial burden for those affected [3]. It is defined as dysfunction of the lymphatic system, resulting in the build-up of lymph fluid in the fatty tissues just under the skin.

There are two types of lymphedema: primary and secondary. Secondary lymphedema is acquired due to another disease or extrinsic factor, such as cancer and it is treatment (surgery or radiation), infection, trauma and parasites.

The diagnosis of lymphedema can be established by both subjective and objective methods [4, 5]. Subjective methods include clinical history and specific questionnaires, while objective methods involve perometry, lymphoscintigraphy, magnetic resonance imaging, computed tomography scan and bioimpedance spectroscopy. Among these, clinical history, specific questionnaires and physical examination are the most commonly used.

Linear measurement, a method used to determine lymphedema, involves measuring the circumference of the affected and normal limbs. A difference of more than 2 cm is considered significant for diagnosing lymphedema [5]. The advantages of linear measurement include being inexpensive and convenient for outpatient evaluation. However, it is time-consuming, requires user experience and is less sensitive to small-volume changes.

Therapy for lymphedema consists of conservative measures as well as surgical measures [6]. Conservative measures consist of lifestyle modification, exercises including gentle muscle contractions in the affected limb to help drain excess fluid, massage techniques for manual drainage of lymph, compression bandages and garments and use of sequential pneumatic compression stockings. Severe cases or refractory cases may be treated with surgery. Various surgical procedures include lymph node (LN) transplant, lympho-venous anastomosis, creating new drainage pathways and removal of fibrous tissue and skin [6]. Prevention and early detection in at-risk patients form the cornerstone of lymphedema management.

Here, we have evaluated the factors causing lymphedema and its association with treatment modalities in carcinoma cervix, which will help in better management.

## Aims and objectives

### Aim

To determine the incidence of post-treatment lower limb lymphedema in cases of carcinoma cervix and its correlation with demographic and treatment-related factors.

### Primary objectives

- To determine the incidence of lymphedema in carcinoma cervix post-treatment.
- To study the association of treatment modality with lower limb lymphedema.

### Secondary objectives

- To investigate the impact of body mass index (BMI) on the development of lower limb lymphedema post-treatment.

## Methods

### Type of study

Retrospective observational study

### Duration of study

Data collected for the duration of 2 years (from January 2022 to December 2023). A total of 100 patients with carcinoma cervix were included in the study population.

### Inclusion criteria

Patients with carcinoma cervix who have completed their primary treatment, in the form of:

- Surgery followed by radiotherapy (RT)/concurrent chemo-radiotherapy (CCRT)
- Concurrent chemo-RT
- Concurrent chemo-RT + Brachytherapy.

### Exclusion criteria

- Preexisting lower limb edema
- Lower limb vascular disease
- Hypoalbuminemia
- History of surgery or trauma on the lower limb
- Dermatological/autoimmune disorders of the lower limb.

All patients were screened in the OPD during 3-monthly follow-up post-treatment. Patient’s who met the inclusion and exclusion criteria and gave consent were included in the study. A questionnaire (Lower Extremity Lymphedema Screening Questionnaire,2012 by Mayo Foundation for Medical Education and Research) was filled out [7], and linear measurement was used for diagnosing lymphedema. A difference of 2 cm or more in the circumference of the affected limb was considered significant.

Statistical analysis was performed using SPSS 21.0. Odds ratios were calculated by univariate analysis.

## Results

We studied 100 patients, of whom 15 developed lymphedema. The mean age and BMI were 49.15 years (median age = 48 years) and 21.54 kg/m^2^ (median BMI = 22), respectively. Eight patients were younger than 49 years and 7 were older. Lymphedema was classified using The International Society of Lymphology (ISL) classification (Table 1). All 15 cases belonged to Stage 1 lymphedema.

All 15 patients developed lymphedema 5 to 8 months post-treatment. The maximum number of 8 patients developed lymphedema by 6 months, followed by 5 patients by 5 months and one each at 7 and 8 months post-treatment (Table 2).

Three patients underwent surgery (radical hysterectomy + bilateral LN dissection). Of these, 1 received adjuvant chemo-RT and developed lower limb lymphedema 5 months post-treatment. The remaining two received adjuvant external beam RT followed by vaginal brachytherapy (one received one fraction of brachytherapy and the other received three fractions). The patient who received three fractions developed lymphedema 6 months post-treatment.

Among the remaining 97 patients, 13 developed lower limb lymphedema. All received external beam RT with concurrent therapy.

Squamous cell carcinoma comprised 91% of the cases, while adenocarcinoma was 8% and clear cell carcinoma was 1%. Lymphedema occurred in 67% of stage III, 22% of stage II and 11% of stage I patients. Almost all (97%) patients had received exclusive RT.

The odds ratio for external beam RT was 1.03, for age was 1.04 and for stage was 1.4. Thus, an association was found between lymphedema and age, stage of the disease and external beam RT. There was no association found between lymphedema and surgery or BMI (Table 3).

## Discussion

The incidence of lymphedema was 15% in the present study. Similar findings were found by Dessources *et al* [8] in a review article stating that the rates of treatment-related lower extremity lymphedema ranged from 10% when assessed retrospectively to 41% when prospectively assessed using objective metrics. A similar incidence was found by Sung *et al* [9] (15%) in a study to determine adjuvant therapy as a risk factor for lymphedema.

In a meta-analysis Hu *et al* [10] found BMI, age, International federation of Gynaecology and Obstetrics (FIGO) stage, RT, LN dissection and the number of LNs removed are the main risk factors for lower extremity lymphedema after cervical cancer treatment. Similarly, we have found an association of lower limb lymphoedema with age, FIGO stage and method of treatment. However, as cases of surgical management are few in our study we were not able to analyse the effect of LN dissection on lower limb lymphoedema.

Japanese study Kuroda *et al* [11] studied 100 patients who had a median follow up period of 6 months, assessment done using ISL criteria for staging. They found an incidence of 33% in post treatment case. Major risk factors for lower limb lymphoedema being BMI ≥25 kg/m^2^, pelvic and para-aortic lymphadenectomy and adjuvant RT which were similar to our findings [11].

Nakamura *et al* [12] studied 97 patients and found 9.5% incidence in post CCRT patients, and 51% in post-surgery + adjuvant RT which were not seen in our study due to less number of operable cases of Ca Cervix.

Halaska *et al* [13] used a self-reporting questionnaire in 60 patients to determine the incidence of lymphedema (25.80%). BMI and RT were the significant risk factors in the cases [13]. Our study had no association of BMI with lymphedema but EBRT showed a positive association.

Mendivil *et al* [14] studied lower limb lymphedema in gynecological malignancies and in a subset of cervical cancer of 30 patients found that the incidence was 3% and association was found with BMI, FIGO stage and RT. The findings of this were similar to the current study [14].

Chang *et al* [15] found a threefold increased risk of lower limb lymphoedema with adjuvant RT in gynaecological malignancies using a propensity risk score matching analysis. Similarly, we have found radiation therapy to be a risk factor for lymphedema in lower limb [15]. A GOTIC study by Hu *et al* [10] also found a higher incidence of lymphedema in patients receiving RT and combined chemotherapy and radiation therapy.

A meta-analysis by Bona *et al* [16] analysed 23 studies and found that the main factors associated with lymphedema included an extension of lymphadenectomy, number of LNs removed, removal of circumflex iliac LNs and adjuvant RT. Other factors associated with lymphedema included cellulitis, lympho-cyst formation, increased age, invasive LN staging, higher BMI and insufficient physical activity. Similarly, in the current study, we found that CCRT, age and stage had an association with lymphedema [16].

Zhou [17] studied 98 patients by self-reporting method and detected lymphedema in 33.67% of cases. Association of lymphedema was found with BMI, age and FIGO stage [17].

Our study is the first of its kind in western India analysing the risk factors of ca cervix patients with an aim to improve their quality of life. We have evaluated multiple factors and followed them up over a period of more than 6 months. However, we have limitations in this being a retrospective study and also the accuracy of retrospective diagnosis of lymphoedema. Also, surgical intervention forms a negligible number in our sample size.

## Conclusion

We found that there is an association of lower extremity lymphedema in cervical cancer post-treatment with age, FIGO stage and method of treatment in our study. However, BMI was not associated with lower limb lymphedema post treatment. Further prospective studies with larger sample sizes and better means of quantifying lymphoedema would be necessary for developing effective to reduce the risk of lower extremity lymphedema during the treatment of Carcinoma Cervix cases.

## Conflicts of interest

The authors have no conflicts of interest to disclose.

## Funding

No financial funding was received for this study.

## Author contributions

All authors were directly involved in the planning, management and follow-up of the patients included in the study.

## Figures and Tables

**Table 1.** Patient demographics.

Patient characteristics	*N* = 100	Average
Age (years)	24–75	49.15 (Median – 48)
BMI (kg/m^2^)	12–33	21.54 (Median- 22)
Stage	I II III	11 22 67

**Table 2.** Table showing stage wise treatment given in carcinoma cervix and number of cases developed lymphedema in various stages.

Stage of Ca Cervix	Treatment taken	Number of patients (*n* = 100)	Lymphedema (*n* = 15)
I (*n* = 11)	Surgery + PORT	3	2
	CCRT + ICR	8	-
II (*n* = 22)	CCRT only	11	-
	CCRT + ICR	11	3
III (*n* = 67)	CCRT only	24	1
	CCRT + ICR	43	9
Total - 100		Total - 100	Total - 15

**Table 3.** Table showing analysis of ODDs ratio for all variables

Variables	Odds ratio	95% C.I.	
		Lower	Upper
Histology	0.396	0.070	2.236
Grade	0.765	0.345	1.697
Stage	1.300	0.447	3.782
Age	1.044	0.964	1.131
BMI	0.943	0.751	1.184
EBRT	1.031	0.348	3.054
Brachytherapy	0.222	0.091	0.540
